# Diagnosis and Therapy of Community-Acquired Pneumonia in the Emergency Department: A Retrospective Observational Study and Medical Audit

**DOI:** 10.3390/jcm13020574

**Published:** 2024-01-19

**Authors:** Giorgia Lüthi-Corridori, Andrea I. Roth, Maria Boesing, Fabienne Jaun, Philip E. Tarr, Anne B. Leuppi-Taegtmeyer, Jörg D. Leuppi

**Affiliations:** 1 University Institute of Internal Medicine (UIIM), Cantonal Hospital Baselland, 4410 Liestal, Switzerland; giorgia.luethi-corridori@ksbl.ch (G.L.-C.); andrea.roth@ksbl.ch (A.I.R.); maria.boesing@ksbl.ch (M.B.); fabienne.jaun@ksbl.ch (F.J.); anne.leuppi-taegtmeyer@usb.ch (A.B.L.-T.); 2 Faculty of Medicine, University of Basel, 4056 Basel, Switzerland; philip.tarr@ksbl.ch; 3 University Center for Internal Medicine, Infectious Diseases and Hospital Epidemiology Service, Cantonal Hospital Baselland, 4101 Bruderholz, Switzerland; 4 Department of Patient Safety, Medical Directorate, University Hospital Basel, 4031 Basel, Switzerland

**Keywords:** pneumonia, community acquired, management, audit, Switzerland

## Abstract

Background: Despite advances in therapy, community-acquired pneumonia (CAP) is still associated with significant morbidity and mortality. Several studies conducted in different countries have reported suboptimal adherence to the guidelines. However, there are currently no available data on adherence to CAP guidelines specifically in Switzerland. Objectives: The aim of this study was to audit the quality of diagnosis and therapy of CAP at a Swiss general hospital. Methods: A retrospective, observational, single-center cohort study was conducted, including patients older than 18 years diagnosed with CAP and admitted to a medical ward throughout 2019 without prior antibiotic therapy prescribed by their general practitioner (GP). The baseline characteristics of the patients were analyzed, and the diagnostic workup and treatment were compared to the Swiss guidelines for CAP. Results: A total of 254 patients diagnosed with CAP were included in this study (median age 78 years, 51.6% males). Atypical pneumonia was diagnosed in 4% of patients, while an organism was identified in 33% of cases, with Streptococcus pneumoniae being the most frequently detected pathogen (57%). A chest image was taken in almost all patients. Documentation of respiratory rate was missing in 23% of cases. Procalcitonin was measured in 23.2% of cases. Pneumococcal and legionella urinary antigen testing was performed on approximately 90% of all patients and blood cultures were drawn in approximately 80% of patients. In 39% of cases, arterial blood gas analysis was performed. Guideline adherence for the administration of empiric antibiotics was documented/recorded in 75% of cases. Twelve different antibiotic regimens were administered, and they were mostly amoxicillin/clavulanate with or without macrolides, as suggested by the guidelines. In particular, the use of ceftriaxone was higher (19.7%) compared to the Swiss guidelines. The average length of antibiotic therapy was longer (8.2 days) compared to the guidelines (5–7 days). Oral steroid therapy was administered to 29.1% of patients, including to 75% of those diagnosed with COPD. Conclusion: Overall, guideline adherence was moderately low, especially with regards to the assessment of respiratory rate, performance of arterial blood gas analysis, and sputum collection. Regarding antibiotic therapy, the use of ceftriaxone and the length of antibiotic therapy should be reduced. Further research is needed to identify the reasons for guideline non-adherence, and to find effective measures for the improvement of guideline adherence.

## 1. Introduction

Community-acquired pneumonia (CAP) is one of the most common infectious diseases worldwide. In Switzerland, approximately 65,000 cases occur per year [1]. Despite advances in therapy, morbidity and mortality are still high [2,3]. CAP is responsible for approximately 2.5 million deaths worldwide every year, making it the most common cause of death due to infectious disease both in Switzerland and abroad [1,4]. Moreover, 30,000 patients with pneumonia are hospitalized every year in Switzerland; therefore, the clinical and financial burden of CAP is substantial [4]. A timely initiation of adequate therapy is crucial for improving patient outcomes and reducing the overall burden of CAP [5].

Overall, the most common cause of pneumonia is Streptococcus pneumoniae, with an incidence of approximately 25% of all cases worldwide, followed by Haemophilus influenzae [6]. Detection of the microbial etiology of CAP can be challenging. In multiple CAP studies, no pathogen was identified in the majority of patients who were admitted to hospital [7,8,9,10].

Resistance to antibiotics has increased in recent years [11]. Antibiotic stewardship regarding the treatment of CAP includes elements such as antibiotic de-escalation, shortening the duration of antibiotic therapy, and an early switch from intravenous to oral antibiotics, as well as adherence to the guidelines when it comes to empiric therapy [11]. Moreover, previous studies have showed that adherence to empiric antibiotic guidelines for CAP is associated with a decreased morbidity and mortality [12,13,14], shorter length of hospital stay [15,16] and decreased healthcare costs [17].

Several local, national, and international guidelines regarding the management of CAP have been developed [18,19,20,21,22]. They provide recommendations for the diagnosis and treatment of CAP. Although research studies have indicated that the implementation of CAP guidelines leads to a substantial decrease in morbidity and mortality, discrepancies between up-to-date guidelines and their implementation in healthcare practice have been recorded frequently, which can result in suboptimal care and inefficient use of resources [16,23,24,25,26,27,28,29]. A first step in the solution to fill the gap is the detection and evaluation of deviations from the guidelines. Hospital internal clinical audits, if set up and carried out effectively, are powerful instruments in quality improvement [30,31]. A few audits on the quality of CAP management have been conducted in different countries in recent years, investigating different aspects of CAP management [32,33,34]. In Switzerland, a formal audit has—to our knowledge—not been published so far.

The aim of this retrospective audit was to assess the quality of the diagnostic and therapeutic management of CAP in a Swiss public teaching hospital and the adherence to the national guidelines.

## 2. Materials and Methods

### 2.1. Design and Setting

Our study was conducted in the Cantonal Hospital of Baselland (KSBL), a district general teaching hospital covering a stable population of 280,000 in northwest Switzerland. We undertook a retrospective, observational cohort study.

### 2.2. Study Population

**Figure 1:**
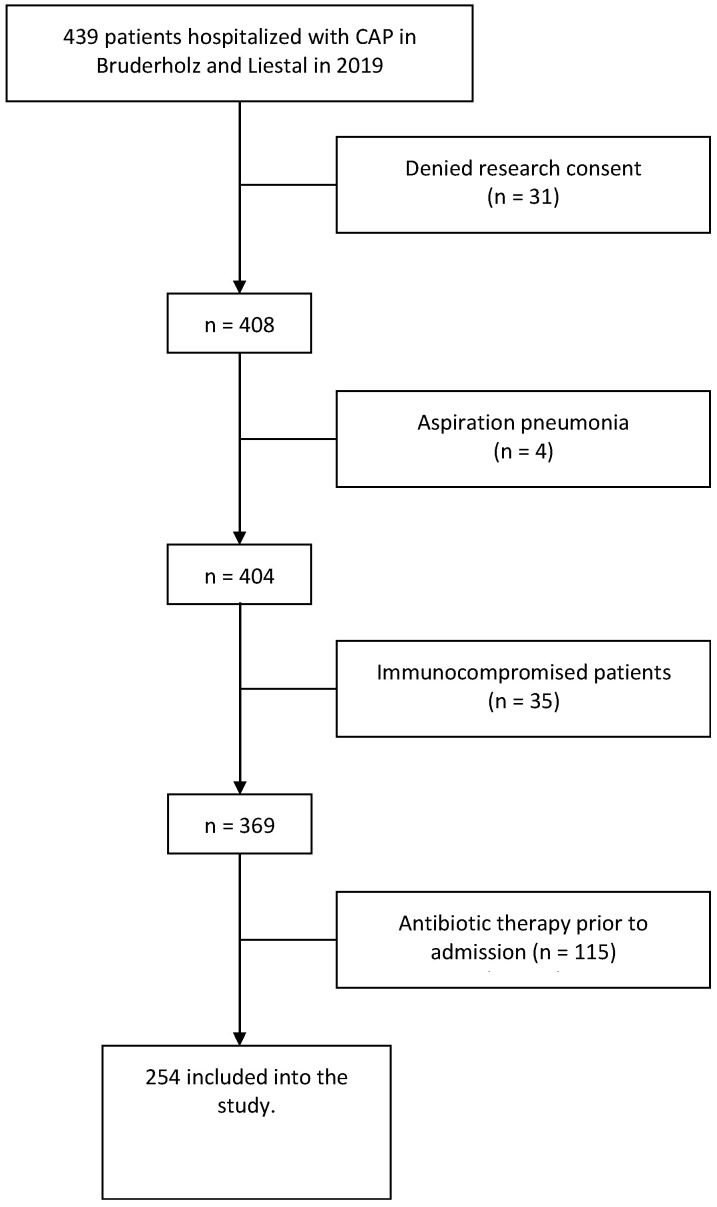
Flow chart of the screening and enrolment of patients.

Clinical routine data of all patients older than 18 years hospitalized with CAP between January and December 2019 were evaluated. We employed ICD-10 codes to identify and include patients in our study. The inclusion criteria involved searching the hospital database for individuals admitted via the emergency department. Patient disposition is shown in Figure 1.

### 2.3. Data Collection and Analysis

Demographic information was extracted from the hospital’s information system. The remaining variables were collected manually from the electronic patient records. These parameters included laboratory imaging, treatment data, symptoms, and medical history, as well as patient outcomes. Respiratory multiplex PCR was performed with the BIOFIRE Respiratory Panel.

The diagnostic workup and treatment were compared to the national guidelines issued by the Swiss Society for Infectious Diseases (SSI) [18] and the recommendations provided to the medical residents online by our hospital’s Department of Medicine (“med standards”, University Hospital Basel, editors) [35].

Descriptive statistics were performed using IBM SPSS Statistics 24. For continuous variables, as measures of central tendency, we display the mean and standard deviation (SD) in the case of a normal distribution, and the median with interquartile range in the case of a skewed distribution (assessed with a histogram). For categorical variables, we report absolute and relative frequencies.

### 2.4. Guidelines

We compared the results with the following recommendations of the Swiss guidelines: [18]

Diagnosis of CAP:

- Confirming pneumonia with a chest X-ray of all patients.
- Obtaining two pairs of blood cultures in hospitalized patients.
- Urine antigen testing (legionella and pneumococcal antigen) in hospitalized patients.
- Obtaining sputum culture if it can be processed within 4 h.
- Performing influenza PCR on a respiratory specimen during influenza season (Note that this study was performed prior to the COVID-19 pandemic).
- Additional aspects recommended by “med standards”: [35]
- Peripheral oxygen saturation (SpO2) and respiratory rate should be measured.
- Auscultation and percussion should be performed.
- Hemogram and blood chemistry (including procalcitonin) should be measured.
- Arterial blood gas analysis should be performed.
- Therapy of CAP:
- Empiric therapy according to the guidelines.
- Length of antibiotic therapy is 5 days (at least 2 days after reaching clinical stability), while for severe pneumonia it is 5–7 days (at least 2 days after reaching clinical stability).
- Oral steroids for patients with chronic obstructive pulmonary disease (COPD) or asthma with evidence of obstruction on pulmonary auscultation for 5–7 days.

## 3. Results

### 3.1. Patient Characteristics

A total of 254 patients diagnosed with CAP were included in this study (see Figure 1). Patient characteristics are shown in Table 1.

The median age of the study sample was 78 years old (IQR 66–85) and more than half of the cohort were males (51.6%). Approximately one quarter of all patients were obese (23.6%) and 25% were active smokers at the time of admission. However, smoking status was not documented in 48% of cases. Hypertension was the most common comorbidity (58.7%). Almost a quarter of all patients had COPD (22%), 7.9% had asthma, and 13.8% had any other form of underlying chronic lung disease. With regards to medications before hospital admission, approximately 24.8% of cases used inhaled medication.

### 3.2. Diagnostics and Assessment

The overview of the images performed can be seen in Table 2. In almost all patients (99.6%), a chest image was performed at admission. In most patients (94.1%), a chest X-ray was performed. For 11% of patients, a CT scan was performed at admission. Pulmonary infiltrate was detected in 86.6% of all cases. The determination of infiltration in chest images was conducted by the radiologists who issued the radiology reports.

Blood cultures were obtained from the majority of patients (81.1%). Less than 10% of blood cultures showed the growth of an organism (19 positive out of 206 blood cultures). Urinary legionella antigen testing was performed on 90.6% of patients, of which 3% were positive. The frequency of pneumococcal urinary antigen testing was similar (89.4%), and it was positive more frequently (22.5%).

Sputum microbiology was obtained from 16.5% of patients, showing a growth in culture in approximately one quarter (26.2%). Respiratory multiplex PCR was performed on 4.3% of patients, detecting at least one pathogen in more than half of cases. Table 3 summarizes the performed microbial diagnostics.

In four patients, a bronchoscopy was performed to gain additional information about the pneumonia.

Patient vital signs at admission are shown in Table 4. Peripheral oxygen saturation and heart rate were measured and documented in all patients, with 33% of patients having their oxygen saturation measured while receiving supplemental oxygen. The mean oxygen saturation in patients without supplemental oxygen therapy was 93%, whereas in patients with supplemental oxygen, mean saturation was 94%. Approximately 50% of patients exhibited tachypnoea. However, the respiratory rate was not documented in 59 cases (23.2%).

At admission, one third (34.7%) showed tachycardia, while 2.4% exhibited bradycardia. The mean blood pressure was 137/75, with one third (33.6%) presenting hypertension and 7.1% being hypotensive.

A complete blood count and blood chemistry including CRP were performed at admission on all patients, while procalcitonin was measured in less than one quarter of patients (23.2%). In 5.9% of patients, procalcitonin was measured at admission. The median CRP value at admission was 129 mmol/L, with an interquartile range from 54.5 to 222.0. The mean leucocyte count was 12.0 G/L (IQR 8.9–15.5).

The recommended arterial blood gas analysis was performed on less than half of patients. Alkalosis was documented in 27% of patients and hypoxemia in more than 40%. One fifth also had hypocapnia. In almost three quarters of all COPD patients, arterial blood gas analysis was performed.

### 3.3. Therapy

The empiric antibiotic therapy is shown in Table 5. Most patients were treated with amoxicillin/clavulanate, either alone (56.7%) or combined with clarithromycin or clindamycin (12.2% in total). Ceftriaxone was the second-most frequently administered drug, with 19.7% alone and 5.1% combined with a macrolide. A penicillin allergy was documented in the medical records for 10.6% of patients. The choice of empiric antibiotic therapy was adherent to the guidelines in approximately 75% of patients.

**Figure 2:**
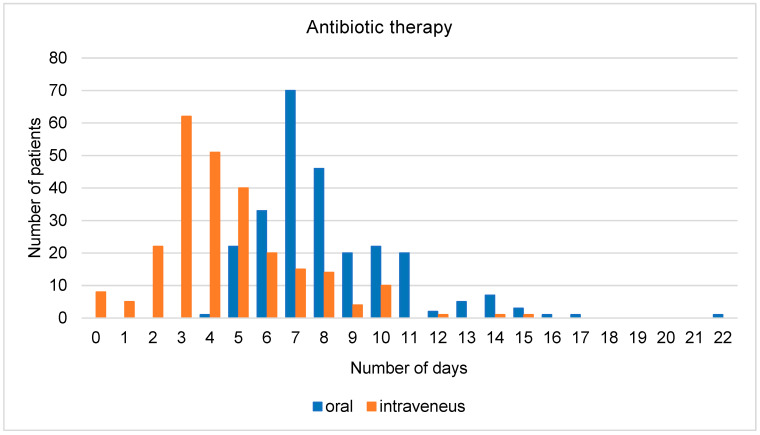
Total number of days of antibiotic therapy (oral/intravenous).

The average duration of overall antibiotic therapy was 8.2 days and the average duration of intravenous antibiotic therapy was 4.3 days (Figure 2). The average time from admission until the administration of the antibiotics was 5 h, while in nine patients, it took more than 24 h. When only counting those patients who received antibiotics within 24 h, the average time was 3.7 h. For 39% of patients, antibiotic therapy was modified at least once during hospitalization. The most common reason for a change was the adaptation of antibiotic therapy to the identified underlying pathogen. Other common reasons were the de-escalation of initial broad-spectrum therapy, unavailable oral equivalent when switching from intravenous to oral, and lack of response to the therapy.

**Figure 3:**
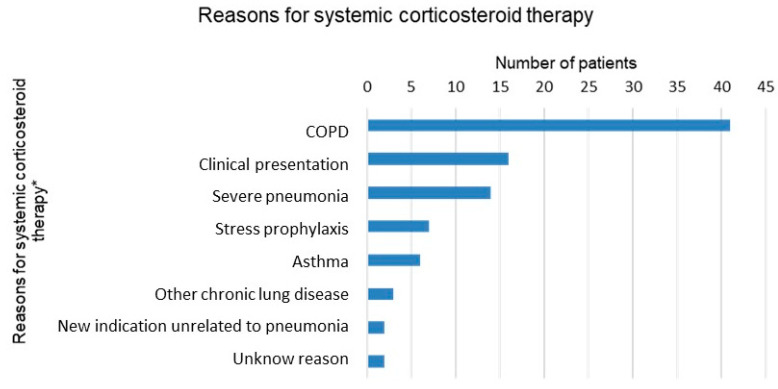
Reasons for systemic corticosteroid therapy documented in patients’ records. * Multiple answers per patient possible.

Systemic corticosteroid therapy was co-administered with antibiotics for 29.1% of all patients. The average length of corticosteroid therapy was 4.9 days (SD, (range)). Among the possible reasons for the administration of systemic corticosteroids (Figure 3), by far the most common was COPD, followed by clinical presentation (obstructive breath sounds on pulmonary auscultation) and severity of the pneumonia. Of the 56 patients with COPD, three quarters received systemic corticosteroids. In addition, in three quarters of all patients transferred to the intensive care unit (ICU), corticosteroids were administered.

## 4. Discussion

In this retrospective observational cohort study of patients hospitalized with community-acquired pneumonia (CAP) at a Swiss general teaching hospital in 2019, we identified moderately low adherence to national and local CAP guidelines, particularly in the assessment of respiratory rate, arterial blood gas analysis, and sputum collection. Regarding the diagnostic workup of CAP, adherence to guideline recommendations was achieved by conducting blood cultures and urinary antigen testing in almost all patients. Additionally, vital signs (but not respiratory rate) and blood tests, such as hemogram and blood chemistry (excluding procalcitonin), were collected in the majority of cases. Notably, a chest image was obtained for all patients except one, demonstrating adherence to guideline recommendations regarding these diagnostic procedures. In terms of therapy, the choice of antibiotic adhered to the guidelines for 75% of patients. However, the average duration of antibiotic therapy was 8.2 days, exceeding the recommended duration. Systemic corticosteroids were administered to almost one third of all patients. Moreover, most patients underwent physical therapy and inhalation therapy.

### 4.1. Comparison of Findings to the Guidelines: Narrative Discussion

#### 4.1.1. Diagnosis of CAP

- Confirming pneumonia with a chest X-ray on all patients

In our audit, the first point of assessment, which required the confirmation of pneumonia with a chest X-ray for all patients, indicated a high level of guideline adherence; in fact, in almost all patients (99.6%), a chest image (either X-ray or CT scan) was performed at the time of presentation. There was only one patient in which no image was documented in patients’ records. It is worth mentioning that the national CAP guidelines also indicate a possible alternative to the chest X-ray, namely, a thoracic ultrasound in cases where experienced staff are available and X-ray is not possible. In our case, thoracic ultrasound was not performed, most likely due to a lack of availability of adequately trained and experienced staff. The diagnosis in the case not documented by chest imaging was based on the assessment of clinical symptoms, lung auscultation, laboratory results (elevated laboratory values), and the positive results of a urine test (pneumococcal antigen detected). Recent review articles have concluded that urine antigen detection tests have shown a high specificity, suggesting that a positive result indicates the causative pathogen of CAP in clinical practice [36,37,38]. Interestingly, in 13% of cases who underwent a chest image, no infiltrate was recorded. Nevertheless, the diagnosis of pneumonia was made on clinical grounds. In conclusion, while our audit shows a high adherence to chest imaging guidelines for CAP, the specificity of urinary antigen tests and the frequency of cases where chest images did not document any pulmonary infiltrates underscores the need to balance diagnostic rigor with minimizing resource utilization in the management of CAP.

- Obtaining two pairs of blood cultures in hospitalized patients

We observed that blood cultures were obtained in a substantial proportion of cases, i.e., in 80% of patients. This finding aligns closely with the results of other audits conducted in similar healthcare settings. For instance, at James Paget University Hospital in the United Kingdom, blood cultures were obtained for 84.2% of cases [32]. Likewise, at Sligo University Hospital in Ireland, the pre-interventional and post-interventional rates for blood culture collection were 84.4% and 62.5%, respectively [33]. The most common reason for not obtaining blood cultures, in 20% of cases within our study, despite being recommended in the guidelines, likely includes that antibiotic therapy had already been started. The 10% rate of positive blood cultures in our study is consistent with the published literature and contributes to the ongoing debate about the cost, merit, and clinical implications of obtaining blood cultures in patients hospitalized with CAP [39,40,41].

- Urine legionella and pneumococcal antigen testing

The assessment of urinary antigen testing, specifically for the detection of pneumococcal and legionella antigens in hospitalized patients, revealed a high compliance rate, approximately 90%, in the present study compared to other European audits. As evidenced by a study conducted in Italy by Costantini et al. in 2012, adherence to both urinary antigen tests was reported at 55% for all patients [29]. Conversely, a study in the United Kingdom by Fahimi et al. demonstrated a markedly lower compliance rate, with less than 20% of physicians adhering to the testing protocol [32]. In Ireland, both pre- and post-intervention results exhibited compliance rates below 20% and up to 40%, respectively [33]. Among the diagnostic tests for CAP, urine antigen tests have been widely considered useful due to their simplicity of collection and the rapidity of the test results [36,42,43]. However, the variability in terms of guideline compliance highlights the need for harmonizing clinical urinary antigen testing practices.

- Obtaining sputum culture if it can be processed within 4 h

It is noticeable that sputum microbiology was only performed on 16.5% of all patients, even though it is recommended to conduct sputum microbiology in all patients admitted to hospital if it can be processed within 4 h. At our hospital, which has an on-site 24-h diagnostic laboratory, a sputum sample can be processed well within 4 h at all times. One possible explanation for the low number of sputum cultures could be the clinical observation that obtaining a sputum sample can be challenging, as it requires patient cooperation and the ability to produce a suitable specimen. Additionally, patients may not fully understand the importance of sputum testing, leading to reluctance or non-cooperation. Other audits also show low numbers of obtaining sputum microbiology. In Ireland, it was just under 20% and approximately 30% in the pre-intervention and post-intervention groups, respectively [33]. In the audit of El Fahimi et al., the adherence to local guidelines regarding sputum collection was just over 20% in 2015 [32]. In addition, despite the guidelines suggesting to obtain sputum culture in all patients, the clinical value of sputum cultures in the management of CAP remain controversial [44]. The published literature suggests that the diagnostic yield of sputum cultures is clearly lower than 50% [45,46]. In a recent meta-analysis encompassing 24 studies and involving 4533 adult CAP patients, a bacterial pathogen was identified in only 36% of sputum samples [47]. However, when good-quality sputum specimens were selected, the test had a summary sensitivity of 0.69 and specificity of 0.91 for detecting Streptococcus pneumoniae.

- Performing influenza PCR during influenza season

Throughout the whole 2019 year, influenza PCR was performed on 50% of all patients. The influenza season in the time frame of our study lasted from approximately 1 January 2019 to 20 April 2019 [48]. During the influenza season, influenza PCR was performed on 72.6% of hospitalized CAP patients. Potential reasons for the lower-than-recommended influenza PCR testing might be related to a low local influenza prevalence or based on clinical presentation, for instance, patients who presented with symptoms that were not strongly indicative of influenza may not have been prioritized for testing. This observation warrants closer examination in the context of optimizing diagnostic practices and the timely detection of influenza cases.

- Respiratory multiplex PCR panel only in selected cases

A respiratory panel examination (respiratory multiplex PCR) was performed on 4.3% of patients. Multiplex PCR assay panels allow faster and comprehensive detection of a wide range of clinically relevant markers. In recent years, numerous multiplex PCR assays have been introduced to the market, and the guidelines have recommended the procedure in certain indications [49]. The hospitalizations who were evaluated in this audit took place in 2019 when this newer form of pathogen identification was not yet recommended in the guidelines.

- Peripheral oxygen saturation (SpO2) and respiratory should be measured

The initial assessment of oxygen saturation upon patient admission was consistently recorded for all individuals within our study. However, compliance with the comprehensive documentation of oxygen requirements throughout the hospitalization period was limited. As far as it was documented, 48% of all patients needed oxygen therapy at some point during hospitalization. It is striking that more than half of all patients in this population were tachypneic at the time of presentation. However, the respiratory rate was not documented for 59 patients, so we may have overestimated the prevalence of tachypnoea at presentation, considering that healthcare workers are more likely to assess and document the respiratory rate in tachypneic patients compared to patients with a normal respiratory pattern. The respiratory rate was also moderately documented in other published audits. In clinical audits at the European Gaza Hospital in 2015 and 2016, the respiratory rate was not documented for 73% of patients [34]. Respiratory rate is relatively easy and quick to assess and it is an important risk parameter for predicting in-hospital mortality [50]. It is also included in various severity scores such as CRB-65 and qSOFA score. Thus, it should be assessed and documented more comprehensively.

- Complete blood count and blood chemistry (including CRP and PCT) should be measured

At our hospital, the inflammation parameters CRP and leucocytes are determined routinely for all patients presenting at the emergency department with respiratory symptoms, so unsurprisingly there were no missing values. On the other hand, procalcitonin is not routinely measured; in our population, PCT was available for only 23% of patients. A meta-analysis by Kamat et al. in 2020 found a pooled sensitivity and specificity of procalcitonin of 0.55 and 0.75, respectively, for detecting bacterial pneumonia [51]. However, a meta-analysis by Schuetz et al. published in 2018 showed that the measurement of procalcitonin is associated with a reduction in antibiotic exposure as well as a significantly reduced 30-day mortality [52]. Another meta-analysis by Pepper et al. in 2019 showed an increased survival and shorter antibiotic duration associated with PCT-guided antibiotic discontinuation but noted that there was a low certainty and high risk of bias [53]. In a 2018 study by Huang et al., PCT-guided therapy did not show a shortened duration of antibiotic therapy compared to usual care [54]. However, the duration of antibiotic therapy was already very short, with an average of 4.2 days in the PCT-guided group and 4.3 days in the usual group. Our audit showed an average duration of antibiotics of 8.2 days. Thus, implementing a procalcitonin-guided antibiotic treatment at our hospital could potentially facilitate shortening the length of antibiotic therapy and therefore also potential adverse events associated with antibiotics.

- Auscultation and percussion should be performed

Pulmonary examination findings were documented for 246 patients (96.9%). In four patients (1.6%), pulmonary examination was not feasible due to an uncooperative patient. In another four patients (1.6%), the examination findings were not documented. Auscultation and percussion are easy assessments that help physicians complete the clinical assessment and differential diagnosis of a patient in addition to laboratory parameters; therefore, it is highly recommended to perform a chest examination during the clinical assessment of any patient with possible CAP.

- Arterial blood gas analysis should be performed

Evaluating arterial oxygen levels plays a crucial role in the initial assessment of patients diagnosed with severe CAP. Hypoxemia is associated with potential respiratory failure, ICU admission, and mortality, indicating the severity of organ dysfunction [55,56,57]. Identifying arterial hypoxemia, moreover, has immediate treatment implications, such as supplemental oxygen administration and closer clinical monitoring. Consequently, measuring arterial oxygenation is a crucial quality indicator in the initial management of CAP individuals. In our cohort, a blood gas analysis was performed on 46.5% of all patients and 73% of patients with COPD. These moderate numbers could likely be improved; however, informal discussions with emergency room physicians in our hospital suggest low enthusiasm for blood gas analysis in clinically stable CAP patients who are in no or little respiratory distress.

- Additional comments on the diagnosis of CAP

The use of clinical risk scores, such as CRB-65, PSI, SOFA, and qSOFA score, was rarely documented. These scores may help estimate the severity of CAP and distinguish whether patients can be treated as outpatients or should be hospitalized. Moreover, recent studies have demonstrated the significant predictive value of risk scores, not only in terms of mortality but also of other outcomes such as length of hospital stay and rehospitalization [58,59,60,61,62,63]. Improvements in calculating and documenting risk scores are in order. Regarding complications documented in the discharge report, more details are showed in Table A1 in the appendix.

#### 4.1.2. Therapy of Community-Acquired Pneumonia

- Empiric therapy according to the guidelines

In this study, we recorded the administration of 12 distinct antibiotic regimens within our sample. When compared to the Swiss guidelines [18], in 75% of cases, the antibiotic therapy was administered according to the guidelines. There are interesting discordances in empirical CAP antibiotic therapy guidelines, where the Swiss guidelines advocate ceftriaxone only as an alternative therapy [18], in contrast to the German guidelines designating it as a first-line antibiotic [19].

Notably, our study highlighted instances where certain patients received a single dose before the antibiotic therapy was changed. We attribute this most likely to standard practices in the emergency department, where ceftriaxone was initiated as an empirical therapy before a definitive diagnosis was made. Furthermore, our investigation revealed an over-administration of piperacillin/tazobactam. The Swiss guidelines recommend piperacillin/tazobactam only for patients with severe pneumonia (i.e., patients admitted to ICU) and risk factors for resistance, in combination with a macrolide (clarithromycin or azithromycin). In our study, we found that piperacillin/tazobactam was also administered to patients with moderate severity pneumonia (e.g., stable vital signs, no evidence of sepsis, admission to regular hospital ward) without macrolide combination therapy.

Other published audits have found low adherence to local CAP guidelines. In the UK, the National Audit Report from 2018 to 2019 showed an adherence of 58% to local antibiotic guidelines [64]. An audit of James Paget University Hospital (UK) showed a compliance of 86% with antibiotic-prescribing guidelines [32]. The audit at Sligo University Hospital (Ireland) showed an increase in overall compliance with local CAP guidelines, from 21.6% to 62.5% (*p* < 0.001), after implementing an intervention bundle in 2019 [33]. At the European Gaza Hospital, 81% of patients received antibiotics that were in line with the local guidelines [34]. In summary, adherence to the guidelines concerning empiric antibiotic therapy for CAP could be improved at our hospital. Firstly, the use of ceftriaxone should be reduced. Furthermore, the prescription of broad-spectrum antibiotics such as piperacillin/tazobactam should be reserved for cases with documentation of a clear indication. Finally, it is worth considering that patients with severe influenza pneumonia should not receive steroids. To provide more clarity on the impact of diagnostic results on therapy and its duration, we would like to highlight that of the 21 patients in our sample who tested positive for influenza A, only 3 received steroids, due to Addison’s disease, COPD, and asthma, respectively.

- Duration of antibiotic therapy: 5 days (at least 2 days after reaching clinical stability), for severe pneumonia, 5–7 days (at least 2 days after reaching clinical stability)

Regarding the duration of antibiotic therapy, the average time of total antibiotic therapy in our dataset was 8.2 days. The Swiss guidelines recommend a duration of 5 days (or 2 days after reaching clinical stability). In severe pneumonia, a duration of 5–7 days is recommended. The average duration of antibiotic therapy in our group was therefore longer than recommended. The published audit from Ireland revealed longer-than-recommended antibiotic duration in their pre-intervention group but shorter-than-recommended duration in the post-intervention group [33].

- Systemic corticosteroids for patients with COPD or asthma with evidence of bronchial obstruction, for 5–7 days

This recommendation is difficult to evaluate retrospectively. Just under 30% of all patients received systemic corticosteroids during their hospital stay. There were several reasons documented why patients received systemic corticosteroids. The most frequent reason was COPD; however, evidence of bronchial obstruction was not documented in any instance. Overall, 75% of patients with COPD received systemic corticosteroids therapy, while 21 patients (28.3%) who received systemic corticosteroids had neither COPD nor asthma. Some patients had reasons unrelated to pneumonia documented for receiving systemic corticosteroids, such as stress prophylaxis during long-term or chronic corticosteroid therapy preceding hospital admission. In our population, severe pneumonia was documented as an indication for steroids in 14 cases. The average length of systemic corticosteroids was just under 5 days, which was shorter compared to the guidelines. Based on our informal discussions with staff internal medicine physicians, we noted considerable awareness to keep steroid treatment in CAP as short as possible. In summary, due to insufficient documentation, we are unable to accurately evaluate the appropriateness of corticosteroid use in the majority of CAP patients in our study.

### 4.2. Limitations and Future Research

The strengths of our study include that findings reflect everyday real-life clinical practice at a mid-size university-affiliated teaching hospital in Switzerland. Our study has a number of limitations, mainly related to its retrospective and observational design. In the setting of missing data, we are unable to verify whether data were obtained but not documented, i.e., missing data were treated as “not obtained”, potentially leading to an underestimation of the quality of care provided in our hospital. Moreover, a comparison of our data with published audit data collected in other hospitals seems difficult due to local differences in medical practice and other differences.

Our sample size was limited to exclusively include hospitalized patients diagnosed with CAP in the emergency room. Potential avenues for future research include exploring the care of immunocompromised patients with pneumonia, hospital-acquired pneumonia, and CAP patients diagnosed and treated by their general practitioner in an outpatient setting and/or those patients who were admitted to hospital at a later stage. Finally, one parameter to determine the length of antibiotic therapy and the switch from intravenous to oral antibiotics is clinical stability. In this study, data on clinical stability were not collected, because it was neither explicitly documented nor possible to collect retrospectively from patients’ records. Therefore, conducting a prospective study is suggested as a more robust method to precisely assess the optimal duration of antibiotic administration for patients.

Early CAP diagnosis may play an important role in timely and appropriate treatment. Our study has highlighted that the diagnosis of pneumonia is not always straightforward to attain. Future studies evaluating the effectiveness of computerized systems for automated diagnosis are desirable. Furthermore, the guidelines should take these issues into account and suggest an additional approach that considers alternative diagnostic options based on the comprehensive evaluation of the patient’s data, including laboratory tests, clinical examination, and symptoms.

## 5. Conclusions

This audit demonstrates room for improvement at our hospital as regards the diagnosis and therapy of community-acquired pneumonia, when taking the national guidelines as our reference.

Firstly, measuring respiratory rate at admission and documenting risk factors should be improved. While urinary antigen testing and obtaining blood cultures were performed more consistently compared to other European settings, there is room for improvement in obtaining sputum microbiology. Arterial blood gas analysis could be performed more frequently, especially in COPD patients. Also, procalcitonin might be measured more often.

Regarding empiric antibiotic therapy of CAP, the use of ceftriaxone could be reduced, and broad-spectrum antibiotics might be administered more carefully. The length of antibiotic therapy could be reduced. Finally, potential indications for systemic corticosteroids in patients with CAP should be documented more carefully.

Our teaching hospital employs numerous physicians from different educational backgrounds. We believe that, to a certain extent, our findings can be generalized to other Swiss and EU hospitals. Further research is needed to identify the reasons for guideline non-adherence, and to find effective measures for the improvement of guideline adherence in the management of hospitalized patients with CAP.

## Figures and Tables

**Table 1:** Patient characteristics.

	All (n = 254)	Missing n (%)
Demographics		
Age, median (IQR)	78 (66–85)	
Male, n (%)	131 (51.6)	
Housing situation prior to admission		
Private home, n (%)	219 (86.2)	
Care facility, n (%)	35 (13.8)	
BMI, median (IQR)	25.7 (22.3–30.1)	92 (36.2)
Obesity (BMI ≧ 30), n (%)	60 (23.6)	19 (7.5)
Smoking status		122 (48)
Lifelong non-smoker, n (%)	42 (31.8)	
Current smoker, n (%)	33 (25)	
Former smoker, n (%)	57 (43.2)	
Comorbidities		
Hypertension, n (%)	149 (58.7)	
Other cardiovascular disease, n (%)	144 (56.7)	
COPD, n (%)	56 (22)	
Asthma, n (%)	20 (7.9)	
Other chronic lung diseases, n (%)	35 (13.8)	
Oxygen therapy at home, n (%)	15 (5.9)	
Diabetes mellitus type 1 and 2, n (%)	45 (17.7)	
Active cancer, n (%)	21 (8.3)	
Severe chronic kidney disease (KDIGO G3b or higher), n (%)	34 (13.4)	
Hospital outcomes		
Length of stay in day LOS, median (IQR)	6.5 (5–9)	
Admission to ICU/IMC, n (%)	24 (9.4)	
Death within 30 days, n (%)	3 (1.2)	
Death within 1 year, n (%)	23 (9.1)	
Readmission to KSBL within 30 days, n (%)	24 (9.4)	

Abbreviations: IQR interquartile range, BMI body mass index, COPD chronic obstructive pulmonary disease, KDIGO Kidney Disease: Improving Global Outcomes, ICU intensive care unit.

**Table 2:** Images performed.

Images Performed, n = 253, (99.6%)	n (%)
Imaging	253 (99.6)
Chest X-ray at admission	239 (94.1)
Chest CT all	49 (19.3)
CT at admission	28 (11.0)
CT during hospitalization	24 (9.4)
Infiltrate detected	219 (86.6)

Abbreviations: CT computed tomography.

**Table 3:** Microbial diagnostics.

Type of Test			Result	n (%)
Blood cultures			performed, n (%)	206 (81.1)
			positive, n (%)	19 (9.2)
			no growth, n (%)	187 (90.8)
Urinary antigen testing				
	Legionella antigen		performed, n (%)	230 (90.6)
			positive, n (%)	7 (3.0)
			negative, n (%)	223 (97.0)
	Pneumococcal antigen		performed, n (%)	227 (89.4)
			positive, n (%)	51 (22.5)
			negative, n (%)	176 (77.5)
Influenza				
	All patients		performed, n (%)	127 (50.0)
		Influenza A	positive, n (%)	20 (15.7)
		Influenza B	positive, n (%)	0 (0)
	During influenza season (n = 168) ^a^		performed, n (%)	122 (72.6)
Sputum microbiology			performed, n (%)	42 (16.5)
			growth of a respiratory pathogen, n (%)	11 (26.2)
			no growth, n (%)	31 (73.8)
Respiratory multiplex PCR			performed, n (%)	11 (4.3)
			pathologic, n (%)	6 (54.5)

^a^ Influenza season: 1 January 2019–20 April 2019 (53) and 28 September 2019–31 December 2019 (20). Abbreviations: PCR polymerase chain reaction.

**Table 4:** Vital and laboratory parameters at the time of admission.

Vital Signs	
Respiratory rate measured, n (%)	195 (76.8)
Respiratory rate, mean (SD)	22.15 (±5.91)
Tachypnoea (respiratory rate >20), n (%)	100 (51.3)
Oxygen saturation measured n (%)	254 (100)
Without supplemental oxygen therapy, n = 221 (87%), mean % (SD)	92.59 (±4.79)
Hypoxaemia (SpO2 < 90%), n (%)	38 (17.2)
With supplemental oxygen therapy n = 33 (13%), mean % (SD)	93.64 (±3.26)
Hypoxaemia (SpO2 < 90%), n (%)	3 (9.1)
Supplemental oxygen administered during hospitalization, n (%)	122 (48)
Blood pressure measured, n (%)	246 (96.9)
Systolic blood pressure (mmHg), mean (SD)	131.65 (±24.34)
Diastolic blood pressure (mmHg), mean (SD)	75.57 (±15.65)
Hypertension (systolic blood pressure > 140 mmHg)	85 (33.6%)
Hypotension (systolic blood pressure < 100 mmHg)	18 (7.1%)
Heart rate measured, n (%)	254 (100)
Heart rate, mean (SD)	92.61 (±18.95)
Tachycardia (heart rate ≥ 100 bpm), n (%)	88 (34.6)
Bradycardia (heart rate < 60 bpm), n (%)	7 (2.76)
Body temperature measured, n (%)	243 (95.7)
Body temperature °C, mean (SD) ^c^	37.70 (±0.98)
Fever (≥38.5° C), n (%)	59 (23.80)
Hypothermia (<36.0 °C), n (%)	6 (2.36)
CRP measured, n (%)	254 (100)
Value at admission, median (IQR)	129 (54.5, 222.0)
Elevated at admission (value ≥ 5 mg/L), n (%)	248 (97.6)
Highest value during hospitalization, median (IQR)	161.5 (91.5, 248,8)
Leucocytes measured at admission, n (%)	254 (100)
Value at admission, median (IQR)	12.0 (8.9, 15.5)
Elevated at admission (value > 10.5 10^9^/L), n (%)	161 (63.39)
PCT measured, n (%)	58 (22.8)
Value at admission, median (IQR)	0.29 (0.10, 0.91)
Elevated value at admission (value ≥ 0.25 μg/L), n (%)	31 (53.5)
Value measured during hospitalization, median	0.23 (0.16, 0.58)

Abbreviations: SD standard deviation, SpO2 saturation of peripheral oxygen, mmHg millimeters of mercury, bpm beats per minute, °C degrees Celsius, CRP C-reactive protein, PCT procalcitonin.

**Table 5:** Empiric antibiotic therapy.

Type of Antibiotic Therapy	n (%)
Amoxicillin/clavulanate only	144 (56.7)
Amoxicillin/clavulanate + Clarithromycin	29 (11.4)
Amoxicillin/clavulanate + Clarithromycin + Clindamycin	1 (0.4)
Amoxicillin/clavulanate + Clindamycin	1 (0.4)
Piperacillin + Tazobactam	6 (2.4)
Ceftriaxone only	50 (19.7)
Ceftriaxone + Clarithromycin	12 (4.7)
Ceftriaxone + Azithromycin	1 (0.4)
Cefuroxime	4 (1.6)
Cefepime	1 (0.4)
Levofloxacin	4 (1.6)
Clarithromycin only	1 (0.4)
Choice of empiric antibiotic therapy according to Swiss guidelines	191 (75.2)
Length of antibiotic therapy (days)	
Total number of days of antibiotic therapy, mean (SD)	8.2 (±2.5)
Number of days of intravenous antibiotic therapy, mean (SD)	4.3 (±2.4)
Number of days of macrolide therapy ^a^ (n = 57), mean (SD)	4.1 (±3.4)
Time to antibiotics	
All patients ^b^, mean (SD) hours from admission to emergency department to first antibiotic dose administered	5.0 (±9.3)
When received within 24 h (n = 245), mean (SD)	3.7 (±4.2)
Allergy to antibiotics	
Penicillin allergy, n (%)	27 (10.6)
Cephalosporin allergy, n (%)	2 (0.8)
Fluoroquinolone allergy, n (%)	3 (1.2)

Abbreviations: SD standard deviation. ^a^ Clarithromycin and azithromycin. ^b^ Missing data of 2 patients.

## Data Availability

All data generated during this study were analyzed and the results were included in this article. The data presented in this study are available on reasonable request from the corresponding author. The data are not publicly available due to restrictions on data privacy.

## Appendix A

**Table A1:** A: Complications documented in the discharge report, n (%).

Type of Complication Documented	n, (%)
Pleural effusion ^a^	55 (21.7)
Sepsis	5 (2.0)
ARDS	0 (0)
Empyema	1 (0.4)
Cardiac complications	45 (17.7)
Thromboembolic complications	2 (0.8)
Acute kidney injury (at least AKIN I)	52 (20.5)
Electrolyte disorders	48 (18.9)
Elevated liver enzymes	18 (7.1)
Neurological complications	14 (5.5)
Fall	17 (6.7)
Syncope	3 (1.2)

Abbreviations: AKIN Acute Kidney Injury Network. ^a^ Missing data of 1 patient.
